# Evaluating Strategies to Reduce Ruminal Protozoa and Their Impacts on Nutrient Utilization and Animal Performance in Ruminants – A Meta-Analysis

**DOI:** 10.3389/fmicb.2019.02648

**Published:** 2019-11-15

**Authors:** Xiaoxia Dai, Antonio P. Faciola

**Affiliations:** Department of Animal Sciences, University of Florida, Gainesville, FL, United States

**Keywords:** fiber digestion, lipid, methane production, plant secondary metabolites, ruminal fermentation

## Abstract

Several studies have evaluated the effects of complete or partial ruminal protozoa (RP) inhibition; however, to this date, no practical suppressant has been identified and used in large scale. This meta-analysis quantitatively evaluates the effectiveness of multiple strategies on inhibiting RP numbers and their influence on ruminal fermentation and animal performance. This study compared 66 peer-reviewed articles (16 manuscripts for complete and 50 manuscripts for partial RP inhibition that used supplemental phytochemicals and lipids, published from 2000 to 2018, to inhibit RP *in vivo*. Data were structured to allow a meta-analytical evaluation of differences in response to different treatments (complete RP inhibition, phytochemicals, and lipids). Data were analyzed using mixed models with the random effect of experiment and weighted by the inverse of pooled standard error of the mean (SEM) squared. Supplemental phytochemicals and LCFA had no effects on inhibiting RP numbers; however, supplemental MCFA had a potent antiprotozoal effect. Both complete and partial RP (supplemental phytochemicals and lipids) inhibition decreased methane production, total tract digestibility of OM and NDF, and ruminal NH_3_-N concentration and increased propionate molar proportion. Methane production, molar proportions of acetate and propionate, total tract NDF digestibility were affected by the interaction of treatment (supplemental phytochemicals and lipids) and RP numbers. Supplemental phytochemicals and lipids can be effective in reducing methane production when RP numbers is below 7 Log_10_ cells/mL, especially by supplemental saponins, tannins, and MCFA. In terms of animal performance, supplemental tannins could be recommended to control methane emissions without affecting animal performance. However, their negative effects on total tract digestibility could be a drawback when feeding tannins to ruminants. The negative effects of supplemental lipids on milk fat composition should be considered when feeding lipids to ruminants. In conclusion, ruminal protozoa play important roles on methanogenesis, fiber digestion, and ruminal NH_3_-N concentration, regardless of experimental diets and conditions; supplemental phytochemicals and lipids can be effective on reducing methane production when RP numbers is below 7 Log10 cells/mL. Among these partial RP inhibition strategies, supplemental tannins could be recommended to control methane production.

## Introduction

Ruminal protozoa (RP) were first described by Gruby and Delafond (1843) and contribute to ruminal fermentation, having both positive and negative impacts on animal performance. Several studies have evaluated the effects of complete or partial RP inhibition; however, to this date, no practical suppressant has been identified and used in large scale. Ruminal protozoa make up less than 0.01% of the microbial cells in the rumen (Williams and Coleman, 1992). Though few in numbers, ruminal protozoa are relatively large compared to bacteria (5–250 μm long), consisting of 5–50% of the microbial mass in the rumen (Williams and Coleman, 1992; Sylvester et al., 2005). Ruminal protozoa are responsible for substantial microbial protein turnover due to predation of ruminal bacteria (Williams and Coleman, 1992). Complete inhibition of RP has been done to study the roles of RP and complete RP inhibition was suggested to increase microbial protein supply by 30% and reduce methane production by up to 11% (Newbold et al., 2015). However, reduction in feed digestibility has been reported to be likely the main drawback of complete RP inhibition. Because it is not practical, complete RP inhibition is not recommended under farm conditions (Hristov et al., 2013). Partial RP inhibition methods, such as supplemental phytochemicals (essential oils, saponins, and tannins) and lipids [medium-chain fatty acids (MCFA) and long-chain fatty acids (LCFA)], have been recommended as alternative strategies of complete RP inhibition. Because inhibiting RP by supplemental phytochemicals and lipids are practical and could be potentially beneficial to animal production (Williams and Coleman, 1992; Patra and Saxena, 2010).

Thirty-one percent of 76 *in vivo* experiments have demonstrated a concomitant reduction in RP numbers and methane production, and nearly all of these experiments tested lipids as methane mitigation strategies (Guyader et al., 2014). It also has been suggested that saponins mitigate methanogenesis mainly by reducing RP numbers, and condensed tannins act by reducing RP numbers and by direct negative effects on methanogens, whereas essential oils could have a direct negative effect on methanogens (Cieslak et al., 2013). It also has been shown that changes in RP numbers have a linear relationship with changes in methane production associated with supplemental saponins (*r* = 0.69), tannins (*r* = 0.55), and essential oils (*r* = 0.45, Patra, 2010). Lipids and phytochemicals are thus currently the most common additives used to control RP numbers and methane production in ruminants.

Recently, many reviews have been published on plant extract or plant secondary, e.g., saponins, tannins, and essential oils as rumen modifier, but they have been primarily focused on the changes on ruminal fermentation (Wallace et al., 2002; Calsamiglia et al., 2007; Hart et al., 2008; Kamra et al., 2008), ruminal microbial population (Patra and Saxena, 2009), and also their relationship with methanogenesis (Patra, 2010). Supplemental lipids were also reviewed regarding their effects on methane emission, digestibility, ruminal fermentation and lactation performance in cattle (Patra, 2013) and compared between cattle and sheep (Patra, 2014). However, very few have evaluated multiple strategies concomitantly and evaluated the relationship of the change in RP numbers with changes on methane production, ruminal fermentation, nutrient utilization, and animal performance when phytochemicals and lipids were fed to ruminants. Moreover, evaluation of partial or complete RP inhibition and their effects on methane production, ruminal fermentation, nutrient utilization, and animal performance have not been carefully evaluated. Meanwhile, the effectiveness of partial RP inhibition strategies (lipids and phytochemicals) have not been vastly studied.

This meta-analysis quantitatively evaluated the effectiveness of multiple strategies to either completely or partially suppress RP numbers. Therefore, the focus was not only on RP roles, but also on the effectiveness of different strategies to reduce RP numbers. The objectives of this meta-analysis were to: (1) evaluate the effectiveness of different partial RP inhibition strategies (lipids and phytochemicals); (2) evaluate the effects of complete and partial RP inhibition strategies on ruminal microbial fermentation, nutrient utilization, and performance; and (3) evaluate changes on ruminal microbial fermentation, nutrient utilization, and animal performance in partial RP inhibition related to the treatments (phytochemicals and lipids), or RP numbers or their interaction. Our hypotheses were: (1) both complete and partial inhibition of RP numbers would reduce methane production; (2) complete RP inhibition would not be recommended because the negative effects would outweigh the benefits of it; and (3) effectiveness of partial RP inhibition strategies would differ, and partial RP inhibition could potentially improve ruminal fermentation and animal performance; however, these would differ between RP inhibition strategies.

## Materials and Methods

### Data Collection

The database searched included publications from 2000 to 2018, reporting *in vivo* data from experiments published in the English language, in which RP count numbers were measured to systematically review different strategies on controlling RP numbers in the past 20 years. To access publications, the editorial platforms of the US National Library of Medicine National Institutes of Health through PubMed^1^ and the ISI Web of Science^2^ were searched with the following keywords: ruminant, defaunation; ruminant, lauric acid, protozoa; ruminant, myristic acid, protozoa; ruminant, coconut oil, protozoa; ruminant, long-chain fatty acid, protozoa; ruminant, oilseed, protozoa; ruminant, tannins, protozoa; ruminant, saponins, protozoa; ruminant, essential oil, protozoa. The search aimed to identify publications with experiments that were suitable for further exploration. Response of interest included RP numbers, ruminal bacterial count number, methane production, ruminal fermentation (total volatile fatty acids, molar proportion of acetate, propionate, butyrate, and ammonia nitrogen concentration), total tract digestibility [dry matter (DM); organic matter (OM); crude protein (CP) and neutral detergent fiber (NDF); dry matter intake (DMI), milk yield and milk composition (CP, fat, lactose]; and body weight gain (BWG) and responses of interests were collected together with pooled standard error of the mean (SEM).

### Inclusion Criteria

The initial criteria for inclusion of the experiment were that treatment interventions included complete and partial inhibition of RP and RP numbers was reported as cells/mL. The method used to control the RP numbers had to be described for each selected publication.

Searched works in the literature were screened for duplicates and then suitability for inclusion initially by reading the abstract to check that the experiment was conducted regarding controlling RP numbers and the controlling methods were described. Then the materials and methods portion of the publication was read to exclude experiments in which treatments were not implemented as previously described.

### Experiments Included

**FIGURE 1:**
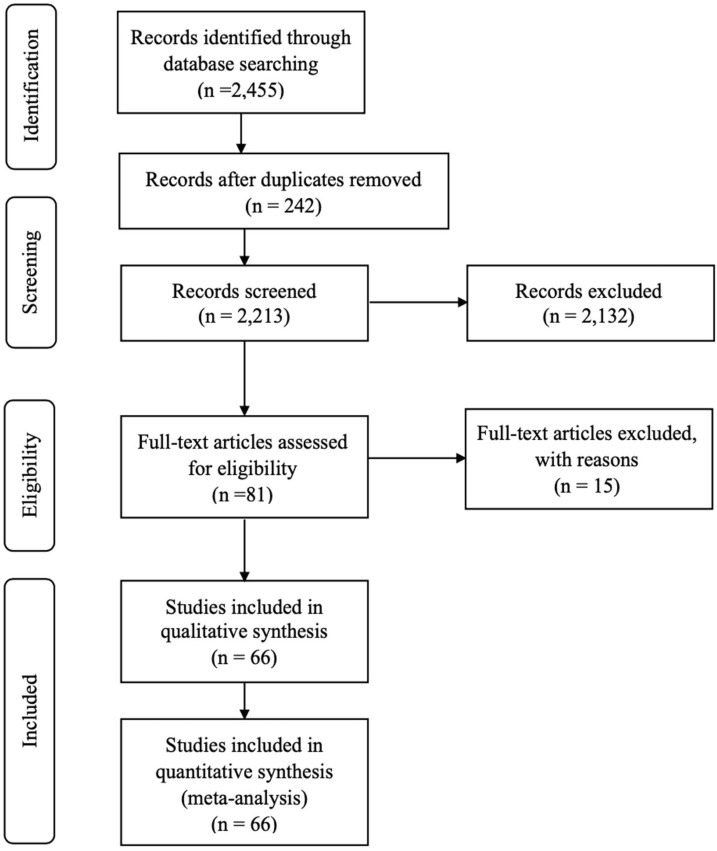
PRISMA flow diagram of the systematic review from initial search and screening to final selection of publications to be included in the meta-analysis. The 66 selected articles for inclusion in the meta-analysis contained multiple experiments; therefore, 87 experiments were used.

Figure 1 depicts a Prisma diagram (Moher et al., 2009) of the flow of data collection for the meta-analysis. After the initial search and screening, 81 publications including multiple experiments were assessed for eligibility. From those, 15 publications were excluded because of the following reasons: publications using 18S rRNA sequencing to determine protozoa concentration; publications in which one treatment that combined more than one RP inhibition strategy; publications in which there were no control group. Even though both microscopy and 18S rRNA sequencing allowed identification of dominant members of the ciliate communities and classification of the RP community, however, microscopy is a highly accurate method for evaluation of total numbers or relative abundance of different RP genera in a sample (Kittelmann et al., 2015). The current meta-analysis was interested in RP numbers and thus 18S rRNA sequencing data were removed from the dataset.

The final database contained 66 publications with 87 experiments and a total of 232 treatment means depending on the response variable were included in the meta-analysis. The list of published papers used is presented in Supplementary Table 1.

Among these publications, experiments belonged to complete inhibition (16 publications, 21 experiments, 45 treatments) consisted of experiments testing effects of completely inhibit RP numbers. The strategies used to completely inhibit RP numbers included: isolated immediately after birth; washed rumen technique; dosing chemical reagent in the rumen (sodium 1- (2-sulfonatooxyethoxy) dodecane; sodium lauryl sulfate). Experiment belonged to phytochemicals (29 publications, 37 experiments, 103 treatments) consisted of experiment testing effects of phytochemicals (tannins, saponins, and essential oil) as protozoa inhibition strategies, in which essential oil included essential oil, garlic oil, peppermint, cinnamaldehyde, eugenol, anise oil, capsicum oil, diallyl disulfide, plant; tannins included plant (e.g., *Kobe lespedeza*, lupiness seeds, *Vaccinium Vitis ideaea* L.), condensed tannin, *Acacia meamsii* extract, grape marc, black currant; and saponins included alfalfa extract, plant powder, sarsaponin, *Maca hyocotyls*, *Sapindus saponaria*, *Quillaja saponaria* extract, *Yucca schidigera* extract, tea saponin. Experiment belonged to lipids (24 manuscripts, Nexp = 29, Ntrt = 84) consisted of experiments testing effects of lipids (LCFA and MCFA) as protozoa inhibition strategies, in which LCFA included linseed, rapeseed, sunflower seed, whole cottonseed, Linola (C18:2), Nulin (C18:3), whole soybeans, algal meal, soybean oil; and MCFA included coconut oil, lauric acid, myristic acid, and sodium laurate.

The data structure and the percentage of experiments that reported specific responses of interest in the selected experiments used for the meta-analysis included: large ruminants (dairy cattle, beef cattle; 65%) and small ruminants (sheep; 35%); methane production (53%); ruminal bacteria (23%); ruminal VFA concentration (total VFA: 96%, acetate: 95%, propionate: 95%, butyrate: 95%); ammonia N (NH_3_-N) concentration (79%); total tract digestibility (DM: 41%, OM: 56%, CP: 42%, and NDF: 57%); Milk yield: 34%; Milk composition (protein: 31%; fat: 31%; lactose: 31%); body weight gain (BWG; 27%). Descriptive statistics of the data included in the meta-analysis was presented in Table 1 based on the strategies on controlling RP numbers. Milk yield and compositions in the present meta-analysis were only from dairy cattle. Descriptive statistic of data included in the meta-analysis based on different animal categories presented in Supplementary Table 2.

**TABLE 1:** Statistical description of the diet and animal characteristics in the data set used for the meta-analysis.

	**Defaunation**			**Phytochemicals**			**Lipids**		
**Item**	***N***	**Mean**	***SD***	***N***	**Mean**	***SD***	***N***	**Mean**	***SD***
**Diet composition, %**									
DM	17	69.5	26.7	58	68.4	19.1	39	67.1	19.0
OM	22	90.9	3.02	76	91.4	4.30	57	92.9	1.93
NDF	33	38.5	11.6	93	35.5	11.4	73	32.4	8.92
CP	29	15.4	3.13	91	17.1	4.00	75	16.6	2.67
**Rumen microorganisms, log cells/mL**									
Protozoa	18	3.02	2.82	103	5.94	1.19	84	5.84	0.71
Bacteria	7	10.7	5.62	40	10.0	2.19	10	11.7	3.95
Methane production, g/kg DMI	16	32.4	13.1	39	24.9	5.40	41	21.8	9.03
**Ruminal fermentation**									
pH	28	9.15	14.6	102	7.10	7.66	67	7.50	9.45
Total VFA concentration, mM	44	88.9	29.1	99	108.8	23.3	83	102	23.7
**Molar proportion, %**									
Acetate	42	68.6	6.45	99	64.9	6.21	83	62.8	6.69
Propionate	42	21.1	12.3	99	21.9	8.40	83	22.9	8.97
Butyrate	42	11.0	13.7	99	12.2	8.76	83	12.2	9.55
NH_3_-N, mM	34	12.1	13.7	96	9.36	8.62	57	8.17	10.3
**Total tract digestibility, %**									
DM	11	58.7	10.56	45	64.2	5.29	44	63.4	7.56
OM	17	66.9	7.62	66	67.4	6.45	56	66.8	4.89
CP	17	67.8	13.14	58	65.0	10.55	36	65.4	7.72
NDF	15	51.4	6.97	67	53.5	8.64	52	47.3	10.42
**Animal performance**									
Milk yield, kg/d	–	–	–	36	29.41	6.19	44	30.16	5.95
**Milk composition, %**									
Protein	–	–	–	36	4.28	4.61	38	3.85	4.53
Fat	–	–	–	36	4.21	4.63	38	4.34	4.48
Lactose	–	–	–	36	5.38	4.25	36	5.44	4.24
**DMI, kg/d**									
Sheep	10	0.92	0.39	23	0.99	0.23	13	0.81	0.12
Dairy	–	–	–	32	19.5	4.52	52	20.3	4.37
Beef	4	3.45	2.26	30	7.25	1.91	12	6.59	1.73
BWG, kg	14	6.27	8.91	22	4.94	8.84	29	2.87	7.04

For the meta-analysis, protozoal and bacterial number (cells/mL) were expressed as Log10 cells/mL. The total VFA and NH_3_-N concentrations were expressed as m*M*. The molar proportion of acetate, propionate, and butyrate was expressed as percentage of total VFA concentration. Dry matter intake and milk yield were expressed as kg/d, milk compositions were expressed as percentage of total milk yield. Methane production was expressed as g/kg DMI to allow interpretation of data from animals with different levels of DM intake.

### Statistical Analysis

Data were analyzed by mixed models with the MIXED procedure of SAS (SAS ver. 9.4, SAS Institute Inc., Cary, NC, United States). Firstly, data were separately analyzed for the difference between RP inhibition strategies [complete and partial inhibition (lipids and phytochemicals)] with the untreated control on ruminal fermentation, nutrient utilization, and animal performance. Then data were analyzed for the difference among different types of phytochemicals (tannins, saponin, and essential oils) and lipids (LCFA and MCFA) within partial RP inhibition groups with the untreated control on ruminal fermentation, nutrient utilization, and animal performance. For experiments with complete RP inhibition, the effects of treatment were compared with untreated control. For experiments with phytochemicals, the effects of inclusion of phytochemicals and type of phytochemical were compared with untreated control. For experiments with lipids, the effects of inclusion of lipids and the type of lipids were compared with untreated control.

Within the partial RP inhibition data, responses of interest were further analyzed by adding treatment, RP numbers and interaction of treatment and RP numbers as fixed effects and experiment identification as random effects to evaluate the changes of response variables that were contributing to the treatments (phytochemicals and lipids), or RP numbers or their interaction; however, it was not intended to generate prediction models. When there was an interaction effect on the response of interest, it was only evaluated how the response variables of interest varied with the interaction; when there was no interaction effect, the interaction was removed from the model and treatment and RP numbers were evaluated together on their effects on the response variables of interest, then RP numbers was removed from the model to evaluate if the coefficient factors and/or statistical significance (only considered if *P* < 0.05) of treatment were changed before and after adding RP numbers or not. The treatments that used phytochemical, and lipids as RP inhibition strategies were considered as “treatment” and the untreated ones were considered as the control.

All mixed models included the random effect of experiment identification, and responses were weighted using the WEIGHT statement in SAS with the inverse of pooled SEM squared (1/SEM^2^) that was centered for each response analyzed as suggested by Wang and Bushman (1999). The slopes and intercepts by experimental identification were included as random effects, and an unstructured variance-covariance matrix (type = un) was performed at the random part of the model (St-Pierre, 2001). When treatment had significant effects on the response of interest, the treatment by experimental identification was also included as random effects. If random covariance of slopes, intercept and treatment were not converged by unstructured variance, a variance component (type = vc) of variance-covariance structure was performed (St-Pierre, 2001). For each analysis, when Cook’s distance was higher than 1, the study was removed from the database in each specific analysis. Outliers were removed when studentized residuals were greater than 2 or less than -2. Statistical significance was considered at *P* ≤ 0.05, with tendencies identified if 0.05 < *P* ≤ 0.10.

## Results

### Effects on Ruminal Bacteria and Methane Production

Complete RP inhibition increased (*P* = 0.01) ruminal bacteria concentration (Log_10_ cells/mL) by 6% while decreased (*P* = 0.01) methane production by 18% (Table 2).

**TABLE 2:** Effects of complete ruminal protozoa (RP) inhibition on ruminal microorganisms, methane production, total tract digestibility, ruminal fermentation, and animal performance.

**Item**	**Control**	**Complete RP inhibition**	**SEM**	***P*-value**
**Rumen microorganisms, Log_10_ cells/mL**				
Protozoa	–	–	–	–
Bacteria	8.33	8.83	0.94	0.01
Methane production, g/kg DMI	33.1	27.2	3.70	0.01
**Ruminal fermentation**				
pH	6.41	6.43	0.11	0.74
Total VFA concentration, mM	88.8	84.5	5.96	0.09
**Molar proportion, %**				
Acetate	67.0	69.0	1.06	0.01
Propionate	18.8	19.7	0.79	0.03
Butyrate	9.75	7.70	0.58	<0.01
NH_3_-N, mM	11.8	7.88	1.68	<0.01
**Total tract digestibility,%**				
DM	60.8	60.1	3.96	0.43
OM	69.2	66.4	2.51	<0.01
CP	74.2	72.2	3.43	0.38
NDF	53.2	48.7	2.12	<0.01
**Animal performance**				
DMI, kg/d				
Sheep	0.78	0.77	0.03	0.79
Dairy	–	–	–	–
Beef	2.27	1.58	0.53	0.67
BWG, kg	0.23	0.23	0.16	0.99

Overall, supplementary phytochemicals had no effects on ruminal bacteria concentration while decreased (*P* < 0.01) methane production by 20% (Table 3). Among these different phytochemicals, compared to control, essential oils had no effects on ruminal bacteria concentration and methane production; however, supplemental saponins and tannins both decreased (*P* < 0.01) ruminal bacteria concentration by about 11% and decreased (*P* < 0.01) methane production by 15% and 20%, respectively (Table 4). Supplemental lipids had no effects on ruminal bacteria whereas decreased (*P* = 0.05) methane production by 15% (Table 5). Among the lipids sources, methane production was decreased by 20% by MCFA whereas only by 9% by LCFA (Table 6).

**TABLE 3:** Effects of phytochemicals on ruminal microorganisms, methane production, total tract digestibility, ruminal fermentation, and animal performance.

**Item**	**Control**	**Phytochemicals**	**SEM**	***P*-value**
**Rumen microorganisms, Log_10_ cells/mL**				
Protozoa	5.87	5.83	0.19	0.22
Bacteria	9.55	9.46	0.35	0.78
Methane production, g/kg DMI	26.1	20.9	0.22	<0.01
**Ruminal fermentation**				
pH	6.35	6.33	0.06	0.15
Total VFA concentration, mM	109	108	3.45	0.91
**Molar proportion, %**				
Acetate	67.2	64.5	1.04	<0.01
Propionate	20.5	21.7	0.61	<0.01
Butyrate	9.89	11.5	0.46	<0.01
NH_3_-N, mM	8.61	8.31	0.72	0.07
**Total tract digestibility, %**				
DM	65.4	65.2	0.75	0.59
OM	67.3	66.6	1.34	0.07
CP	66.2	65.4	2.00	0.12
NDF	54.0	52.9	1.82	<0.01
**Animal performance**				
Milk yield, kg/d	29.3	29.2	1.54	0.83
**Milk composition, %**				
Protein	3.54	3.45	0.12	<0.01
Fat	3.44	3.45	0.12	0.74
Lactose	4.69	4.69	0.05	0.90
**DMI, kg/d**				
Sheep	0.89	1.07	0.07	0.09
Dairy	20.4	19.9	0.76	<0.01
Beef	7.33	7.49	0.64	0.13
BWG, kg	0.24	0.38	0.06	0.14

**TABLE 4:** Effects of different phytochemicals on ruminal microorganisms, methane production, total tract digestibility, ruminal fermentation, and animal performance.

**Items^1^**	**Control**	**Essential oils**	**Saponins**	**Tannins**	**SEM**	***P*-value**
**Rumen microorganisms, Log_10_ cells/mL**						
Protozoa	5.87	5.85	5.76	5.84	0.19	0.37
Bacteria	9.96^a^	9.85^a^	8.85^b^	8.82^b^	0.35	<0.01
Methane production, g/kg DMI	26.1^a^	25.3^ab^	22.2^b^	20.9^b^	1.46	<0.01
**Ruminal fermentation**						
pH	6.35	6.34	6.31	6.32	0.05	0.40
Total VFA concentration, mM	107^b^	107^b^	110^ab^	112^a^	3.55	<0.01
**Molar proportion, %**						
Acetate	71.6^a^	63.6^*c*^	65.4^*b,c*^	69.0^b^	1.21	<0.01
Propionate	20.6^b^	21.9^a^	21.6^a^	21.6^a^	0.65	<0.01
Butyrate	9.98^b^	11.5^a^	10.8^ab^	11.6^a^	0.67	<0.01
NH_3_-N, mM	8.70	8.48	8.14	7.89	0.74	0.18
**Total tract digestibility, %**						
DM	65.3^a^	65.6^a^	65.3^a^	63.7^b^	0.72	<0.01
OM	67.4^a^	67.4^a^	65.6^b^	65.5^b^	1.30	<0.01
CP	66.2^a^	66.4^a^	64.9^a^	61.3^b^	1.97	<0.01
NDF	53.8^a^	54.0^a^	52.0^b^	52.6^b^	1.82	<0.01
**Animal performance**						
Milk yield, kg/d	29.4	29.4	27.9	29.4	1.56	0.55
**Milk composition, %**						
Protein	3.53^a^	3.42^b^	3.51^ab^	3.46^ab^	0.12	<0.01
Fat	3.44	3.48	3.41	3.40	0.12	0.75
Lactose	4.69	4.72	4.64	4.68	0.05	0.29
DMI, kg/d						
Sheep	0.87^b^	1.01^ab^	0.96^ab^	1.20^a^	0.12	0.04
Dairy	20.5	20.2	19.6	20.9	0.91	0.09
Beef	7.32	7.50	7.52	7.48	0.65	0.46
BWG, kg	0.24	0.38	1.84	1.08	1.27	0.47

*^*a*−−*c*^Least squares means within the same row with different superscripts differ (*P* < 0.05).*

**TABLE 5:** Effects of lipids on ruminal microorganisms, methane production, total tract digestibility, ruminal fermentation, and animal performance.

**Item**	**Control**	**Lipids**	**SEM**	***P*-value**
**Rumen microorganisms, Log_10_ cells/mL**				
Protozoa	5.91	5.76	0.14	0.07
Bacteria	10.4	10.3	0.18	0.11
Methane production, g/kg DMI	23.2	19.7	2.10	0.05
**Ruminal fermentation**				
pH	6.33	6.36	0.05	0.18
Total VFA concentration, mM	102	99.3	2.46	0.01
Molar proportion,%				
Acetate	63.5	62.1	1.09	<0.01
Propionate	21.1	22.2	0.66	<0.01
Butyrate	11.6	10.9	0.45	0.05
NH_3_-N, mM	7.17	6.25	0.68	<0.01
**Total tract digestibility, %**				
DM	64.9	63.4	1.58	0.08
OM	68.4	66.7	0.97	0.01
CP	66.6	66.7	1.93	0.80
NDF	48.3	45.6	2.46	0.06
**Animal performance**				
Milk yield, kg/d	30.4	29.6	1.64	0.09
**Milk composition, %**				
Protein	3.13	3.08	0.05	0.03
Fat	3.85	3.55	0.16	<0.01
Lactose	4.77	4.71	0.04	0.06
DMI, kg/d				
Sheep	0.79	0.80	0.05	0.51
Dairy	20.4	19.4	1.13	0.02
Beef	7.28	6.63	0.72	0.13
BWG, kg	0.60	0.60	0.15	0.99

**TABLE 6:** Effects of different lipids on ruminal microorganisms, methane production, total tract digestibility, ruminal fermentation, and animal performance.

**Items^1^**	**Control**	**LCFA**	**MCFA**	**SEM**	***P*-value**
**Rumen microorganisms, log cells/mL**					
Protozoa	5.97^a^	5.94^a^	5.58^b^	0.14	<0.01
Bacteria	10.2	10.9	10.1	0.28	0.14
Methane production, g/kg DMI	23.1	21.1	18.3	2.16	0.10
**Ruminal fermentation**					
pH	6.33	6.37	6.36	0.06	0.40
Total VFA concentration, mM	103^a^	100^a^	98.5^b^	2.45	0.03
**Molar proportion, %**					
Acetate	63.5^a^	62.5^ab^	61.8^b^	1.12	0.01
Propionate	21.0^b^	21.9^ab^	22.5^a^	0.74	0.01
Butyrate	11.6	11.1	10.7	0.48	0.09
NH_3_-N, mM	7.36^a^	6.52^b^	5.98^b^	0.70	<0.01
**Total tract digestibility, %**					
DM	64.9	62.4	63.8	1.56	0.18
OM	68.4^a^	64.9^b^	67.4^a^	0.98	<0.01
CP	66.6	65.9	66.8	1.98	0.83
NDF	48.3	46.7	45.3	2.53	0.13
**Animal performance**					
Milk yield, kg/d	30.5	29.9	29.4	1.71	0.17
**Milk composition, %**					
Protein	3.13	3.09	3.07	0.06	0.06
Fat	3.86^a^	3.54^b^	3.55^b^	0.16	0.02
Lactose	4.77	4.75	4.69	0.04	0.11
DMI, kg/d					
Sheep	0.79	0.82	0.79	0.05	0.22
Dairy	20.5^a^	20.2^a^	18.6^b^	1.21	<0.01
Beef	7.00	6.25	6.94	0.72	0.75
BWG, kg	0.60	0.56	0.62	0.15	0.87

*^*a*,*b*^Least squares means within the same row with different superscripts differ (*P* < 0.05); LCFA, long chain fatty acids; MCFA, medium chain fatty acids.*

### Effectiveness of Supplemental Phytochemicals and Lipids on RP Numbers Inhibition

Based on the current analysis, supplemental phytochemicals (essential oils, saponins, and tannins) had no effects RP numbers (Log_10_ cells/mL; Tables 3, 4). Supplemental lipids tended to decrease (*P* = 0.07) RP numbers compared to the control (Table 5), where supplemental MCFA decreased RP numbers by 6.5% while LCFA had no effects on RP numbers when compared to the control (Table 6).

### Effects on Total Tract Digestibility

Complete RP inhibition had no effects on total tract digestibility of DM and CP but decreased (*P* < 0.01) both total tract digestibility of OM and NDF (Table 2).

Supplemental phytochemicals decreased (*P* < 0.01) total tract digestibility of NDF and tended to decrease (*P* = 0.07) total tract digestibility of OM, however, it had no effects on total tract digestibility of DM and CP (Table 3). Among phytochemicals, supplemental tannins decreased total tract digestibility of DM, OM, CP, and NDF; however, no differences were observed in total tract digestibility of DM, CP, and NDF by supplemental essential oils and saponins when compared to control (Table 4). Supplemental saponins decreased total tract digestibility of OM compared to control (Table 4). Supplemental lipids decreased (*P* = 0.01) total tract digestibility of OM and tended to decrease total digestibility of DM (*P* = 0.08) and NDF (*P* = 0.06) but had no effect on total tract digestibility of CP (Table 5). Among lipids, supplemental LCFA decreased total tract digestibility of OM and no effect was observed by supplemental MCFA when compared to control (Table 6).

### Effects on Ruminal Fermentation and Ammonia Concentration

Complete RP inhibition tended to decrease (*P* = 0.09) total VFA concentration, increased the molar proportion of acetate (*P* = 0.01) and propionate (*P* = 0.03) whereas decreased the molar proportion of butyrate (*P* < 0.01) and NH_3_-N concentration (*P* < 0.01). However, there was no effect of complete RP inhibition on ruminal pH.

Supplemental phytochemicals had no effects on total VFA concentration and ruminal pH; however, it decreased (*P* < 0.01) the molar proportion of acetate whereas increased the molar proportion of propionate (*P* < 0.01) and butyrate (*P* < 0.01; Table 3). The concentration of NH_3_-N tended to reduce (*P* = 0.07) by supplemental of phytochemicals. Among these phytochemicals, supplemental tannins increased total VFA concentration compared to control (Table 4). Supplemental essential oils, saponins, and tannins decreased the molar proportion of acetate whereas increased the molar proportion of propionate (Table 4). The molar proportion of butyrate was increased by supplemental essential oils and tannins. Supplemental lipids decreased (*P* < 0.01) total VFA and NH_3_-N concentration (Table 5). Supplemental lipids decreased the molar proportion of acetate (*P* < 0.01) and butyrate (*P* = 0.05) whereas increased (*P* < 0.01) the molar proportion of propionate (Table 5). Among different lipids, supplemental MCFA decreased total VFA concentration compared to the control, and it also decreased the molar proportion of acetate whereas increased the molar proportion of propionate, but no differences were observed between the control and supplemental LCFA (Table 6). However, both supplemental LCFA and MCFA decreased NH_3_-N concentration (Table 6).

### Effects on DMI, Body Weight Gain, Milk Yields, and Milk Compositions

Complete RP inhibition had no effect on BWG and DMI (Table 2). As milk yield and composition data of complete RP inhibition were not abundant in the current analysis, the effects of complete RP inhibition on milk yield and milk composition were not evaluated.

Supplemental phytochemicals had no effects on BWG, milk yield, milk fat and lactose, but it decreased DMI in dairy (*P* < 0.01) and milk protein (*P* < 0.01; Table 3). Among these phytochemicals, supplemental essential oils decreased milk protein and supplemental tannins increased DMI in sheep compared to the control (Table 4). Supplemental lipids had no effects on BWG but tended to decrease milk yield (*P* = 0.09) and milk lactose (*P* = 0.06). Supplemental lipids decreased DMI (*P* = 0.02) in dairy cows, milk protein (*P* = 0.03), and milk fat (*P* < 0.01; Table 5). Among these lipids, supplemental MCFA decreased DMI in dairy cows but both supplemental LCFA and MCFA decreased milk fat (Table 6).

### Effects of Treatment, RP Numbers, and Their Interaction on Ruminal Microbe, Ruminal Fermentation, and Animal Performance

**FIGURE 2:**
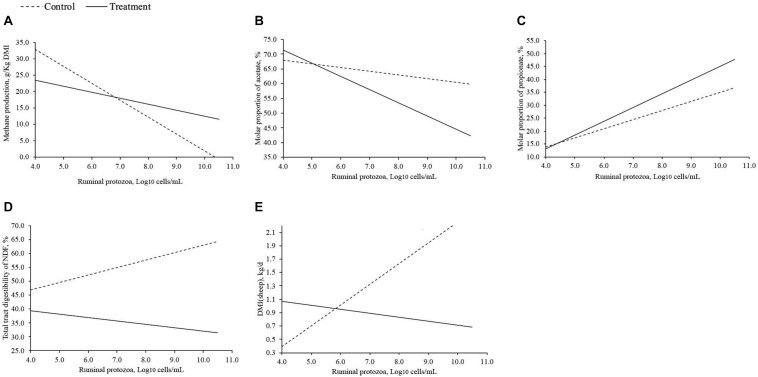
Response of interest that significantly affected by interaction of treatment (phytochemicals and lipids) and ruminal protozoa. **(A)** methane production; **(B)** molar proportion of acetate; **(C)** molar proportion of propionate; **(D)** total tract digestibility of NDF; **(E)** DMI in sheep.

Methane production, molar proportions of acetate and propionate, total tract digestibility of NDF, and DMI in sheep were affected by the interaction of treatment (phytochemicals and lipids) and RP numbers (Log_10_ cells/mL; Table 7). Methane production decreased as RP numbers increased in both control and treatment group, and when the RP numbers (Log_10_ cells/mL) was lower than 7, the treatment decreased methane production; however, treatment had greater methane production compared to the control when RP numbers was greater than 7 (Figure 2A). The molar proportion of acetate decreased as RP numbers increased when RP numbers was lower than 5, molar proportion of acetate was greater in the treatment group. However, when RP numbers was greater than 5, the molar proportion of acetate in the treatment was lower than the control, and there was a greater difference between the control and the treatment as RP numbers increased (Figure 2B). The molar proportion of propionate increased as RP numbers increased in both the control and treatment group, and also the difference between the control and the treatment were increased as RP numbers increased (Figure 2C). Total tract digestibility of NDF in the treatment group was decreased as RP numbers increased; however, it was increased as RP numbers increased in the control group (Figure 2D). Dry matter intake in sheep of the control was increased as RP numbers increased whereas the treatment group decreased as RP numbers increased (Figure 2E), and their difference increased as RP numbers increased when RP numbers was greater than 6.

**TABLE 7:** Effects of treatment (phytochemicals and lipid), ruminal protozoa (RP) and their interaction as predictors on the ruminal microorganisms, methane production, total tract digestibility, ruminal fermentation, and animal performance.

**Items^1^**	**Num**	**Intercept**	***P*-value**	**Treatment^2^**	***P*-value**	**RP^3^**	***P*-value**	**Treatment × RP^4,5^**	***P*-value**
Rumen Bacteria, Log_10_ cells/mL	48	6.71	< 0.01	–0.11	0.67	0.52	< 0.01	NS	NS
	48	9.68	< 0.01	–0.11	0.67	−	−	−	−
Methane production, g/kg DMI	76	53.6	< 0.01	–22.8	0.01	–5.18	< 0.01	3.35	0.05
**Ruminal fermentation**									
pH	167	6.99	< 0.01	–0.02	0.13	–0.11	< 0.01	NS	NS
	167	6.34	< 0.01	–0.003	0.80	−	−	−	−
Total VFA concentration, mM	180	70.5	< 0.01	0.44	0.31	5.89	< 0.01	NS	NS
	180	105	< 0.01	–0.39	0.27	−	−	−	−
**Molar proportion, %**									
Acetate	180	72.8	< 0.01	16.4	0.02	–1.23	0.24	−3.23	<0.01
Propionate	180	−0.21	0.95	–8.12	< 0.01	3.53	< 0.01	1.80	<0.01
Butyrate	180	26.7	< 0.01	0.27	0.03	–2.77	< 0.01	NS	NS
	180	9.92	< 0.01	1.54	< 0.01	−	−	−	−
NH_3_-N, *mM*	151	3.95	0.04	–0.43	< 0.01	0.62	0.05	NS	NS
	151	4.47	< 0.01	–0.44	< 0.01				
**Total tract digestibility, %**									
DM	83	58.8	< 0.01	–0.42	0.26	1.06	0.26	NS	NS
	83	65.0	< 0.01	–0.62	0.08	−	−	−	−
OM	116	44.1	< 0.01	–0.21	0.45	4.06	< 0.01	NS	NS
	116	67.8	< 0.01	–0.89	< 0.01	−	−	−	−
CP	88	63.9	< 0.01	–0.53	0.11	0.43	0.67	NS	NS
	88	66.5	< 0.01	–0.59	0.17	−	−	−	−
NDF	117	36.0	< 0.01	5.44	0.05	2.70	0.12	−1.22	0.03
Milk yield, kg/d	78	18.0	< 0.01	–0.06	0.73	2.04	0.01	NS	NS
	78	29.7	< 0.01	–0.19	0.34	−	−	−	−
**Milk composition, %**									
Protein	72	3.41	< 0.01	–0.07	< 0.01	–0.01	0.52	NS	NS
	72	3.34	< 0.01	–0.07	< 0.01	−	−	−	−
Fat	72	2.31	< 0.01	0.01	0.70	0.21	0.03	NS	NS
	72	3.56	< 0.01	–0.01	0.77	−	−	−	−
Lactose	72	4.33	< 0.01	–0.003	0.86	0.06	0.08	NS	NS
**DMI, kg/d**									
Sheep	36	−0.85	0.15	2.16	< 0.01	0.31	< 0.01	−0.37	<0.01
Dairy	80	10.8	< 0.01	–0.64	< 0.01	1.63	< 0.01	NS	NS
	80	20.3	< 0.01	–0.72	< 0.01	−	−	−	−
Beef	39	4.51	0.01	0.15	0.16	0.41	0.08	NS	NS
	39	7.18	< 0.01	0.06	0.51	−	−	−	−
BWG, kg	48	0.47	0.65	0.10	0.08	0.02	0.92	NS	NS
	48	0.57	< 0.01	0.10	0.06	−	−	−	−

*^1^VFA, voile fatty acids; DM, dry matter; OM, organic matter; CP, crude protein; NDF, neutral detergent fiber; BWG, body weight gain. ^2^Estimate of treatment (phytochemicals and lipids). ^3^Estimate of ruminal protozoa (RP). ^4^Estimate of interaction of treatment and RP. ^5^NS, no significant; –, did not include in the model.*

Ruminal bacteria numbers, pH, milk yield, and milk fat were only affected RP numbers, and the presence of RP had no interference on the impacts of treatment on the three response variables, as coefficient factors and statistical difference of treatment were not altered before and after adding RP numbers in the models (Table 7). Ruminal bacteria, milk yield, and milk fat had a positive relationship with RP numbers whereas pH had a negative relationship with RP numbers. Total VFA concentration was also only positively affected by RP numbers; however, the coefficient factor of treatment on total VFA concentration was shifted from negative (-0.39) to positive (0.44) when RP numbers was added into the model, indicating an interference of RP numbers on the impact of treatment on total VFA concentration (Table 7). Total tract digestibility of OM was negatively affected by treatment (*P* < 0.01); however, the effect of treatment was absent (*P* = 0.50) when RP numbers was added into the model. Instead, total tract digestibility of OM only had a positive relationship with RP numbers (*P* < 0.01) when both treatment and RP numbers as the predictors for total tract digestibility of OM. Milk protein was only negatively affected by treatment (*P* < 0.01). The concentration of NH_3_-N and DMI in dairy were affected by both treatment and RP numbers, and the effect of treatments on the two response variables was independent of RP numbers, as the coefficient factors and statistical significances were not altered by adding RP numbers in the model (Table 7). Both of NH_3_-N concentration and DMI in dairy had negative relationships with treatment while had positive relationships with RP numbers. The molar proportion of butyrate was also affected by both treatment and RP numbers, and the degree of the effects of treatment was decreased by adding RP numbers in the model, where the coefficient factor of treatment on molar proportion of butyrate was decreased from 1.54 to 0.27, observing a positive relationship with treatment whilst a negative relationship with RP numbers (Table 7). Total tract digestibility of DM and CP, milk lactose and DMI in beef animals and BWG were not affected by treatment and RP numbers.

## Discussion

Overall, based on the current study, supplemental phytochemicals had no effects on inhibiting RP numbers and supplemental lipids only had a tendency to inhibit RP numbers, which was a result of supplemental MCFA, e.g., lauric acid, coconut oil, and myristic acid, but not because of supplemental LCFA as it was not different from the control group. Phytochemicals are considered as ruminal modifiers that could improve the efficiency of ruminal fermentation such as enhancing protein metabolism, decreasing methane production, reducing nutritional stress such as bloat, and thus improving animal health and productivity (McIntosh et al., 2003; Patra et al., 2006a; Benchaar et al., 2007). Based on the current study, supplemental phytochemicals had minor impacts on RP numbers, and therefore, supplemental phytochemicals are not recommended as RP suppressing agents.

It has been reported that the effects of tannins on RP numbers are conflicting. Newbold et al. (1997) found that tannins from *S. sesban* foliage did not have anti-protozoa activity. However, Makkar et al. (1995) reported that quebracho tannins reduced the number of total RP, entodiniomorphs, and holotrichs, and the effects were greater on holotrichs. However, anti-protozoa effects of quebracho tannins were not evident in dairy cows (Benchaar et al., 2008). Few studies even reported an increase in RP numbers. Salem et al. (1997) observed a linear increase in RP numbers in rumen fluid of sheep fed an alfalfa-hay based diet by the addition of *Acacia cyanophylla Lindl.* foliage. Therefore, based on the current analysis, tannins have no effect on RP numbers. Compared to RP, other microorganisms may be more sensitive to tannins. Tavendale et al. (2005) found the polymeric fraction of extractable tannins could completely inhibit ruminal methanogens. Tannins also exert inhibitory effects on bacteria (proteolytic and cellulolytic bacteria) and fungi (Tagari et al., 1965; Min et al., 2005; Patra and Saxena, 2009). This was also observed when ruminal bacteria (Log_10_ cells/mL) was decreased by supplemental tannins in the current analysis. The inhibitory activity of tannins against bacteria has been implicated due to the ability of tannins to form complexes with the cell wall and membrane of bacteria causing morphological changes of the cell wall and the extracellular enzymes secreted (Jones et al., 2004; Smith et al., 2005).

Effects of essential oils on RP numbers also have conflicting results in the literature. A blend of essential oils had no effect on RP numbers when fed to dairy cows (Benchaar et al., 2007). Supplementation of diets with cinnamaldehyde to dairy cows (Benchaar et al., 2008) had also no effects on RP numbers. In contrast, steers fed peppermint (*Mentha piperita* L.; containing essential oils) had lower RP numbers, and *Entodinum*, *Isotricha*, and *Diplodinium* (Ando et al., 2003). It has also been observed that essential oils from *S. aromaticum* decreased the number of RP, small entodiniomorphs and holotrichs, but did not affect large entodiniomorphs (Patra et al., 2006b). Therefore, effects of essential oils on inhibiting RP numbers varied with the sources of essential oils, species of RP, as well as species of animals, and thus no effects of essential oil were observed on inhibiting RP numbers in the current analysis. However, essential oils were reported to have direct negative effects on methanogens (Cieslak et al., 2013) and certain ruminal microorganism (Hart et al., 2008). Even though ruminal bacteria concentration was not affected by supplemental essential oils in the current analysis, which may be due to essential oils mainly affecting some specific microorganism, but not all of them (Hart et al., 2008), and the data reported here was overall ruminal bacterial counts, and not specific species.

Therefore, based on the conflicting results of the effects of tannins and essential oils on RP numbers as well as limited effects of essential oils and tannins on RP numbers from the current analysis, we conclude that in general, essential oils and tannins have no effect on reducing RP numbers. Essential oils and tannins, as ruminal modifier, may be affecting other ruminal microorganisms, e.g., ruminal bacteria and methanogens. Meanwhile, different species of RP responded differently to essential oils and tannins. Therefore, future studies should focus on studying the effects of essential oils and tannins on individual species of RP instead of total numbers of RP. This will provide a more accurate evaluation of the effects of essential oils and tannins on RP numbers, and allow better understanding of the mechanism of actions of essential oils and tannins act as ruminal modifiers.

The majority of the research on saponins has been conducted to evaluate it as RP inhibition agents, with the goal of to improve the efficiency of microbial protein synthesis by reducing microbial protein turnover, enhancing protein flow to the duodenum. The anti-protozoal activity of saponins is the most consistent effects among phytochemicals (Patra and Saxena, 2009), which has been reported across different species of ruminants (Lu and Jorgensen, 1987; Newbold et al., 1997; Hristov et al., 1999). However, supplemental saponins had only a numerically reduction (2%) in the current analysis. The inconsistencies of the effects of saponins on RP numbers may be due to different saponins concentrations. This may also result from the large variety of dietary composition, e.g., dietary NDF ranged from 15 to 47% and dietary CP ranged from 12 to 28% in the current meta-analysis. The large variations of diet may affect the effects of saponins on RP. Patra and Saxena (2009) also reported that the composition of the diets and saponins concentration would affect the impacts of saponins on RP numbers.

Lipids can reduce the metabolic activity and numbers of RP (Beauchemin et al., 2009). Medium-chain fatty acids (lauric acid, myristic acid, and related products) were reported to have a potent antiprotozoal effect (Faciola et al., 2013; Faciola and Broderick, 2014). Long-chain fatty acids, including several oils, linseed oil, soybean oil, and fish oil were also inhibitory to RP growth (Machmüller, 2006; Patra and Yu, 2014). However, the effects of lipids on RP numbers varied with the type of fatty acids. Lauric acid was reported to strongly decreased RP numbers compared to myristic and steric acids (Hristov et al., 2012). Patra (2013) also suggested that the type of fatty acids would have different effects on RP numbers. Meanwhile, *Entodinium* and *Epidinium* were more sensitive to linseed oil than *Isotricha* (Ueda et al., 2003; Benchaar et al., 2012). Moreover, when oleic acid or saturated FA were added to the culture medium, *Methanobrevibacter ruminantium*, the most abundant species of methanogens in the rumen, was inhibited (Henderson, 1973), indicating that supplemental LCFA may be more sensitive to methanogens than RP. Therefore, these factors as stated before could explain why supplemental MCFA decreased RP numbers (6.5%) while supplemental LCFA had no effects on RP numbers in the current analysis. The effects of lipids on RP numbers are also dependent on the diets provided to ruminants, as well as the delivery method. It was suggested that high concentrate diets were favorable for reducing the effects of linseed oils on RP numbers (Ueda et al., 2003; Yang et al., 2009; Benchaar et al., 2012). Faciola et al. (2013) observed a strong antiprotozoal activity (∼90%) when lauric acid was given through the ruminal cannula within 2 d of treatment but it only reduced RP numbers by 25% when fed in the TMR, indicating a mode of delivery effect on suppression of RP numbers by lauric acid. Therefore, based on the current analysis, in general, MCFA were more toxic to RP than LCFA. Also, there were more variables that play roles when LCFA are fed to ruminants aiming to inhibit RP numbers.

Ruminal protozoa are not essential to the animal and complete RP inhibition has been used to study the role of RP in the rumen without other dietary interventions (Williams and Coleman, 1992). The current study was intended to evaluate the overall difference between complete and partial RP inhibition strategies (e.g., supplemental phytochemicals and lipids) on methane production, ruminal fermentation, nutrient utilization, and animal performance. For example, our goal was to evaluate if compared to control, a particular method or additive affected the response variables of interest (Supplementary Table 3). We were not intended to evaluate which additive or method was better to control methane production, or increase milk yield, etc. The multiple comparisons were only done within the same category, e.g., within supplemental phytochemicals or within supplemental lipids. At the same time, we were also interested in the differences between partial and complete RP inhibition on methane production, ruminal microbial fermentation, nutrient utilization, and animal performance caused by different treatments (supplemental phytochemicals and lipids) or the effects of RP on these and their interactions between treatments and RP.

Complete RP inhibition reduced methane production (18%), indicating the important role of PR on methanogenesis, as RP could transfer hydrogen to methanogens (Williams and Coleman, 1992), and inhibiting RP decreased methane production. Newbold et al. (2015) also found that complete RP inhibition reduced methane production by up to 11%. Interestingly, we found that partially inhibition of RP or even numerically reduction in RP, were associated with significant reductions in the methane production by supplemental phytochemicals (20%) and lipids (15%), indicating that except for RP, supplemental phytochemicals and lipids themselves also played important roles on methane production. Morgavi et al. (2010) reported that RP could only explain approximately 47% of the variability in methane production, even though a significant linear relationship between methane emission and protozoa concentration were found in another meta-analysis (*r* = 0.96, Guyader et al., 2014).

Based on the current study, we found that there was a treatment (supplemental phytochemicals and lipids) and RP numbers interaction on methane production, indicating that methanogenesis was affected by both treatment and RP. Effects of supplemental phytochemicals and lipids on methane production were dependent on RP numbers. When RP numbers (Log_10_ cells/mL) was lower than 7, supplemental phytochemicals and lipids reduced methane production; however, the difference of methane production between the treatment and the control decreased as RP numbers increased. When RP numbers was higher than 7, methane production in supplemental phytochemicals and lipids groups were greater than the control. These suggested that high RP numbers may compromise the effects of the phytochemicals and lipid on reducing methane production. Because besides RP, other ruminal microorganisms (ruminal bacteria and methanogens) seemed to be more sensitive to supplemental phytochemicals and lipids as stated before, and high RP numbers may interact with the ruminal microbial ecosystem, for example, decrease ruminal bacteria due to their predation of ruminal bacteria (Williams and Coleman, 1992), and thus compromise the effects of treatments on methane production. However, according to the current analysis, the average RP numbers (Log_10_ cells/mL) was 5.64, and only two experiments had RP numbers greater than 7. Therefore, the concentration of RP (Log_10_ cells/mL) in most ruminants would be expected to be lower than 7, and thus supplemental phytochemicals and lipids could be applied as effective methane reducing agents across different experimental conditions, diets, and species of ruminants, especially by feeding saponins, tannins, and MCFA.

The reduction in methane production usually increases the concentration of hydrogen, which could be available to other hydrogen sinks such as propionate, resulting in increased propionate concentration (McAllister and Newbold, 2008; Patra and Saxena, 2010). These could explain why both complete and partial RP inhibition increased the molar proportion of propionate in the current analysis. Meanwhile, a previous meta-analysis (Nozière et al., 2011) has reported that NDF digestibility positively correlates with acetate molar proportion (*r* = 0.95) but negatively with propionate (*r* = −0.94). Both complete and partial RP inhibition decreased total tract NDF digestibility in the current study. Therefore, the decrease in NDF digestibility could partially explain the increase of propionate molar proportion by both complete and partial RP inhibition. Increase of propionate molar proportion by complete RP inhibition indicates the role of RP on ruminal propionate concentration, which may affect methanogenesis and fiber digestion. Meanwhile, there was an interaction of treatment (supplemental phytochemicals and lipids) and RP on the molar proportion of propionate, suggesting that propionate molar proportion was also driven by supplemental phytochemicals and lipids. Increasing RP numbers increased propionate molar proportion, and the difference between the treatment and the control increased with increased RP numbers. This also suggested that an enhanced effect of RP on increasing propionate molar proportion when fed phytochemicals and lipids to ruminants and the reduction of methane production may be the main driving factor to increase propionate molar proportion, as increasing RP decreased methane production.

Conversely, the production of acetate in the rumen results in large quantities of hydrogen and depends on the availability of reducing equivalents such as NAD + (Patra and Saxena, 2010). The high pressure of hydrogen and the high NADH/NAD + ratio in the rumen due to the inhibition of methanogenesis may result in a reduction in acetate production (Miller, 1995). Moreover, NDF digestibility positively correlates with acetate molar proportion (*r* = 0.95; Nozière et al., 2011), and both supplemental phytochemicals and lipids decreased total tract digestibility of NDF. Therefore, both supplemental phytochemicals and lipids decreased the molar proportion of acetate. However, complete RP inhibition increased acetate (+3%), which was similar to Newbold et al. (2015)’s study, that complete RP inhibition slightly increased acetate (+3%). There was also an interaction of treatment (supplemental phytochemicals and lipids). The increase of acetate by complete RP inhibition may not be directly driven by the decrease of methane production and fiber digestion, which may be driven by ruminal bacteria. Complete RP inhibition may be favorable for the growth of acetate-producing bacteria in the rumen.

Complete RP inhibition significantly decreased total tract NDF digestibility, which was also found by Newbold et al. (2015). Reduction of total tract NDF digestibility by complete RP inhibition could be due to the reduction in ruminal NDF digestibility, which reduced by −20% in Newbold et al. (2015)’s study. It has been reported that protozoa play an important role in fiber digestion (Williams and Coleman, 1992), and thus a reduction in total tract fiber digestion was generally found with complete RP inhibition. Therefore, reducing RP numbers is expected to decrease NDF digestibility. This was true when phytochemicals and lipids were not fed to the ruminants. However, it was not the case when treatments were fed to ruminants. Ruminal bacteria and fungi are important for fiber digestion in the rumen (Russell, 2002). However, supplemental phytochemicals and lipids had strong inhibitory effects on bacteria and fungi and increasing RP numbers cannot compensate for the loss of ruminal bacterial and fungal activity toward fiber digestion. The reduction of total tract NDF digestibility by supplemental phytochemicals and lipids also contributed to the decrease of acetate molar proportion and methane production, as less substrate would be available for ruminal microorganism to synthesize acetate and produce hydrogen used for methanogenesis as stated before. Meanwhile, increasing RP numbers decreased methane production, acetate molar proportion and total tract NDF digestibility, and increased propionate molar proportion. This suggested that molar proportion of acetate and propionate, RP numbers, and total tract NDF digestibility were entwined with methane production, which requires further studies to figure out the relationship among them, and thus to assist at migrating methane production by feeding different additives to ruminants. However, when evaluating the relationship, we should also take the effects of treatment into account, as there was an interaction of treatment (supplemental phytochemicals and lipids) and RP numbers on the molar proportion of acetate and propionate, total tract NDF digestibility and methane production. The effects of treatment may bring the confuting effects on the relationship between methane production with acetate, propionate, RP numbers and total tract digestibility.

Both complete and partial RP inhibition decreased ruminal NH_3_-N concentration. Complete RP inhibition reduced ruminal NH_3_-N concentration, which is considered probably the most consistent of the observed effects of complete RP inhibition (Newbold et al., 2015) and appears to be due to the decrease microbial protein breakdown and feed protein degradability in the absence of RP (Williams and Coleman, 1992), suggesting the importance of RP on ruminal NH_3_-N concentration. One of the main effects of RP is the substantial turnover of microbial protein due to predation (Williams and Coleman, 1992), and thus complete RP inhibition renders less turnover of microbial protein and resulting in less accumulation of ruminal NH_3_-N concentration. Less ruminal NH_3_-N concentration means less metabolic energy required for ruminants to inhibit the excess of urea; therefore, more energy could be potentially directed to animal production, especially for diets that are low in nitrogen (Broderick, 2018). Therefore, both complete and partial RP inhibition will be beneficial to the environment and animals in terms of production utilization. Based on the current analysis, ruminal NH_3_-N concentration was affected by both treatment and RP numbers; however, their effects on ruminal NH_3_-N concentration were independent of each other. Supplemental tannins and LCFA had no effects on RP numbers; therefore, the decrease on ruminal NH_3_-N concentration by supplemental tannins and LCFA were mainly attributed to animals fed tannins and LCFA. However, supplemental MCFA decreased RP numbers, and thus the decrease of ruminal NH_3_-N concentration was a result of both MCFA and RP numbers.

Among ruminants, dairy cattle were the most sensitive to supplemental saponins, which was found to reduce DMI and consequently in milk yield. Supplemental tannins and essential oils did not affect animal performance (DMI and milk yield). Supplemental tannins would be thus suggested to control methane emissions without affecting animal performance (DMI, milk yield and milk compositions). However, their negative effects on total tract digestibility would be the drawback when fed tannins to ruminants. Even though a reduction of DMI was found in dairy cattle by supplemental MCFA, milk yield was not affected by MCFA. However, both supplemental LCFA and MCFA reduced milk fat content, which may cause economic losses to producers, as currently milk price is based on milk solid components.

### Future Perspectives About Inhibition of Ruminal Protozoa

Currently, there is a gap in knowledge regarding the relationship of the less abundant RP and animal performance. Instead, the focus has been on the relationship between the total number of RP, not individual taxa, in relationship to animal performance. Milk fat yields and total RP numbers were greater from Holstein cows supplemented with palm oil than those without supplementation (Kirovski et al., 2015), but the specific protozoa involved were not identified. It was reported that *Polyplastron, Entodinium, Isotricha*, and *Dasytricha* persisted in non-lactating dairy cows before and after they were inoculated under subacute ruminal acidosis, but the roles RP play in acidosis has not been fully investigated (Hook et al., 2011). It was also suggested that not all RP species were equally influenced by supplemental phytochemicals (Patra and Saxena, 2009). Meanwhile, RP have complex interactions with ruminal bacteria, methanogens, and fungi. These interactions varied with different species among RP, methanogens, and fungi. Studies on the relationships of individual RP taxa and animal performance would help us to find more effective solutions to mitigate protozoa without interfering on other ruminal microorganisms’ functions. This may be accomplished by culture-independent techniques, as RP cannot grow without bacteria in the culture medium and it is difficult to identify particular RP functions by using culture-dependent techniques (Levy and Jami, 2018; Park and Yu, 2018).

Even though partial RP inhibition strategies affect ruminal fermentation and animal performance as discussed before, studies have provided evidence that the ruminal microbial population is able to adapt to these feed additives over time, especially phytochemicals, which could present a challenge for practical application of these feed additives. Therefore, future studies should focus on identifying the types and doses of these feed additives and the types of diets that would confer positive effects on ruminal microbial populations and fermentation, and thus improve ruminant production.

## Conclusion

Supplemental phytochemicals and LCFA had no effects on inhibiting RP numbers; however, supplemental MCFA had a potent antiprotozoal effect. Both complete and partial RP inhibition decreased methane production, total tract digestibility of OM and NDF and ruminal NH_3_-N concentration and increased propionate molar proportion. Methane production, molar proportion of acetate and propionate, total tract NDF digestibility were affected by the interaction of treatment (supplemental phytochemicals and lipids) and RP numbers. Therefore, reductions in methane production, total tract NDF digestibility, and acetate molar proportion, as well as the increase of propionate proportion by supplemental phytochemicals and lipid depend on RP numbers. Supplemental phytochemicals and lipids can be effective in reducing methane production when RP numbers was below 7 Log_10_ cells/mL, especially by supplemental saponins, tannins, and MCFA. In terms of animal performance, supplemental tannins could be recommended to control methane emissions without affecting animal performance. However, their negative effects on total tract digestibility could be a drawback when fed tannins to ruminants. Meanwhile, the negative effects of supplemental lipids on milk fat composition should be considered when feeding lipids to ruminants. The relationship between RP numbers, molar proportions of acetate and propionate and total tract NDF digestibility with methane production require further study; however, treatment effects should be taken into account when evaluating their relationship. Studying the relationships of individual RP taxa, ruminal fermentation, and animal performance is required to allow the development of more effective methods to control RP and methane production in the future. Meanwhile, the adaptation of the microbial population to feed additives could be a challenge for the application of either complete or partial RP inhibition. Therefore, future studies should focus on identifying the types and doses of these feed additives and their dietary interactions to be more effective as feed additives.

## Data Availability Statement

All datasets generated for this study are included in the article/Supplementary Material.

## Author Contributions

AF: project acquisition. XD and AF: trial and project design and revision of the manuscript. XD: trial implementation, data collection, data analysis (statistics and graphics), data interpretations, and writing the manuscript.

## Conflict of Interest

The authors declare that the research was conducted in the absence of any commercial or financial relationships that could be construed as a potential conflict of interest.

## Footnotes

1 http://www.ncbi.nlm.nih.gov/pubmed

2 http://apps.webofknowledge.com
