# Supplementary material for: Evaluating Strategies to Reduce Ruminal Protozoa and Their Impacts on Nutrient Utilization and Animal Performance in Ruminants – A Meta-Analysis
Source: Front Microbiol. 2019 Nov 15;10:2648. doi: 10.3389/fmicb.2019.02648 (PMC6873214; doi:10.3389/fmicb.2019.02648)
Supplement: Supplementary file 1 [file Table_1.docx]

Supplementary Table 1 Published papers used for the current meta-analysis

| Number | Author | Year | Journal |
| --- | --- | --- | --- |
| 1 | Abubakr et al. | 2012 | J. Saudi Soc Agr Sci.12: 147-154 |
| 2 | Anassori et al. | 2011 | Livest Sci. 142: 276–287 |
| *3 | Ando et al. | 2003 | Livest Prod Sci. 82: 245–248 |
| 4 | Animut et al. | 2008 | Anim Feed Sci Tech. 144: 212–227 |
| 5 | Beauchemin et al. | 2009 | J. Dairy Sci. 92: 2118–2127 |
| 6 | Belanche et al. | 2011 | J. Anim. Sci. 89: 4163–4174 |
| 7 | Belanche et al. | 2015 | FEMS Microbiol Ecol. 91(3), fiu026 |
| 8 | Benchaar et al. | 2006 | J. Dairy Sci. 89: 4352–4364 |
| 9 | Benchaar et al. | 2007 | J. Dairy Sci. 90: 886–897 |
| *10 | Benchaar et al. | 2008 | J. Dairy Sci. 91: 4765–4777 |
| 11 | Benchaar et al. | 2012 | Anim Feed Sci Tech. 178: 139–150 |
| 12 | Bird et al. | 2008 | Aust J Exp Agr. 48: 152–155 |
| 13 | Bryszak et al. | 2018 | J. Dairy Sci. 102: 1257–1273 |
| 14 | Cardozo et al. | 2006 | J. Anim. Sci. 84: 2801–2808 |
| 15 | Carulla et al. | 2005 | Aust. J. Agric. Res. 56: 961–970 |
| *16 | Chung et al. | 2011 | Anim Feed Sci Tech. 166–167: 321–329 |
| 17 | Cieslak et al. | 2012 | Anim Feed Sci Tech. 176: 102–106 |
| 18 | Faciola and Broderick | 2013 | J. Anim. Sci.91:2243–2253 |
| 19 | Faciola and Broderick. | 2014 | J. Dairy Sci. 97: 5088–5100 |
| 20 | Faciola et al. | 2013 | J. Anim. Sci. .91: 363–373 |
| *21 | Fandino et al. | 2008 | Anim Feed Sci Tech. 145: 409–417 |
| 22 | Grainger et al. | 2010 | J. Dairy Sci. 93: 2612–2619 |
| *23 | Guyader et al. | 2017 | J. Dairy Sci. 100: 1845–1855 |
| 24 | Hegarty et al. | 2008 | Br J Nutr. 100: 1220–1227 |
| 25 | Hess et al. | 2004 | Animal Science. 79: 177-189 |
| *26 | Holtshausen et al. | 2009 | J. Dairy Sci. 92: 2809–2821 |
| *27 | Hristov et al. | 2009 | J. Dairy Sci. 92: 5561–5582 |
| *28 | Hristov et al. | 2011 | J. Dairy Sci. 94: 382–395 |
| 29 | Hristov et al. | 2004 | J. Dairy Sci. 87: 1820–1831 |
| *30 | Ivan et al. | 2000 | J Dairy Sci 83: 776–787 |
| 31 | Ivan et al. | 2013 | Br J Nutr. 109: 1211–1218 |
| 32 | Jordan et al. | 2006a | J. Anim. Sci. 84: 2418–2425 |
| 33 | Jordan et al. | 2006b | J. Anim. Sci. 84: 162–170 |
| *34 | Klevenhusen et al. | 2011 | Anim Feed Sci Tech. 166–167: 356–363 |
| 35 | Koenig et al. | 2000 | J. Anim. Sci. 78: 2431–2445 |
| 36 | Lee et al. | 2011 | J. Dairy Sci. 94: 5544–5557 |
| 37 | Lila et al. | 2005 | Asian-Aust. J. Anim. Sci. 18: 1746-1751 |

Supplementary Table 1. Continued.

| Number | Author | Year | Journal |
| --- | --- | --- | --- |
| 38 | Lovett et al. | 2003 | Livest Prod Sci. 84: 135–146 |
| 39 | Machmuller et al. | 2000 | Anim Feed Sci Tech. 85: 41-60 |
| 40 | Mechmuller et al. | 2003a | Reprod. Nutr. Dev. 43: 41–55 |
| 41 | Machmuller et al. | 2003b | Br J Nutr. 90: 529–540 |
| *42 | Martin et al. | 2011 | Proc New Zealand Soc Animal Prod. 71: 243-247 |
| 43 | Moate et al. | 2013 | J. Dairy Sci. 96: 3177–3188 |
| 44 | Moate et al. | 2014 | J. Dairy Sci. 97: 5073–5087 |
| 45 | Mohammed et al. | 2004 | J. Anim. Sci. 82: 1839–1846 |
| 46 | Morgavi et al. | 2008 | Aust J Exp Agr. 48: 69–72 |
| 47 | Morgavi et al. | 2012 | Br J Nutr. 107: 388–397 |
| 48 | Newbold et al. | 2004 | Anim Feed Sci Tech. 114: 105–112 |
| *49 | Nguyen and Hegarty | 2017 | J Anim Physiol Anim Nutr. 101: 984–993 |
| 50 | Ozutsumi et al. | 2005 | Biosci. Biotechnol. Biochem. 69: 499–506 |
| 51 | Panyakaew et al. | 2013 | Animal. 7: 1950–1958 |
| 52 | Pen et al. | 2007 | Anim Feed Sci Tech. 138: 75–88 |
| 53 | Pilajun and Wanapat | 2011 | Livest Sci. 141: 148–154 |
| 54 | Reveneau et al. | 2012 | J. Dairy Sci. 95: 2046–2060 |
| 55 | Rira et al. | 2015 | J. Anim. Sci.93: 334–347 |
| *56 | Staerfl et al. | 2011 | Agric. Ecosyst. Environ. 148: 111–120 |
| 57 | Sahoo et al. | 2005 | J. Dairy Sci. 88: 2027–2036 |
| 58 | Santra and Karim | 2000 | Anim Feed Sci Tech. 86: 251-260 |
| 59 | Schonhusen et al. | 2003 | Arch. Anim. Nutr. 57: 279 – 295 |
| 60 | Soltan et al. | 2009 | Asian J. Anim. Sci. 3: 1-12 |
| 61 | Tekippe et al. | 2011 | J. Dairy Sci. 94: 5065-5079 |
| 62 | Yanez-Ruiz et al. | 2007 | Br J Nutr. 97: 938–948 |
| *63 | Yang et al. | 2007 | J. Dairy Sci. 90: 5671–5681 |
| *64 | Yang et al. | 2010a | J. Anim. Sci. 88: 680–688 |
| *65 | Yang et al. | 2010b | Anim Feed Sci Tech. 158: 57–64 |
| 66 | Zhou et al. | 2011 | Anim Feed Sci Tech. 166–167: 93–100 |

*Reported individual ruminal protozoa species.

**Reference:**

Abubakr, A. R., Alimon, A. R., Yaakub, H., Abdullah, N., and Ivan, M. (2013). Digestibility, rumen protozoa, and ruminal fermentation in goats receiving dietary palm oil by-products. *J. Saudi Soc. Agric. Sci.* 12, 147–154. doi:10.1016/j.jssas.2012.11.002.

Anassori, E., Dalir-Naghadeh, B., Pirmohammadi, R., Taghizadeh, A., Asri-Rezaei, S., Maham, M., et al. (2011). Garlic: A potential alternative for monensin as a rumen modifier. *Livest. Sci.* 142, 276–287. doi:10.1016/j.livsci.2011.08.003.

Ando, S., Nishida, T., Ishida, M., Hosoda, K., and Bayaru, E. (2003). Effect of peppermint feeding on the digestibility, ruminal fermentation and protozoa. *Livest. Prod. Sci.* 82, 245–248. doi:10.1016/S0301-6226(03)00012-5.

Animut, G., Puchala, R., Goetsch, A. L., Patra, A. K., Sahlu, T., Varel, V. H., et al. (2008). Methane emission by goats consuming diets with different levels of condensed tannins from lespedeza. *Anim. Feed Sci. Technol.* 144, 212–227. doi:10.1016/j.anifeedsci.2007.10.014.

Beauchemin, K. A., McGinn, S. M., Benchaar, C., and Holtshausen, L. (2009). Crushed sunflower, flax, or canola seeds in lactating dairy cow diets: Effects on methane production, rumen fermentation, and milk production. *J. Dairy Sci.* 92, 2118–2127. doi:10.3168/jds.2008-1903.

Belanche, A., Abecia, L., Holtrop, G., Guada, J. A., Castrillo, C., de la Fuente, G., et al. (2011). Study of the effect of presence or absence of protozoa on rumen fermentation and microbial protein contribution to the chyme1. *J. Anim. Sci.* 89, 4163–4174. doi:10.2527/jas.2010-3703.

Belanche, A., de la Fuente, G., and Newbold, C. J. (2015). Effect of progressive inoculation of fauna-free sheep with holotrich protozoa and total-fauna on rumen fermentation, microbial diversity and methane emissions. *FEMS Microbiol. Ecol.* 91. doi:10.1093/femsec/fiu026.

Benchaar, C., Lettat, A., Hassanat, F., Yang, W. Z., Forster, R. J., Petit, H. V., et al. (2012). Eugenol for dairy cows fed low or high concentrate diets: Effects on digestion, ruminal fermentation characteristics, rumen microbial populations and milk fatty acid profile. *Anim. Feed Sci. Technol.* 178, 139–150. doi:10.1016/j.anifeedsci.2012.10.005.

Benchaar, C., McAllister, T. A., and Chouinard, P. Y. (2008). Digestion, Ruminal Fermentation, Ciliate Protozoal Populations, and Milk Production from Dairy Cows Fed Cinnamaldehyde, Quebracho Condensed Tannin, or Yucca schidigera Saponin Extracts. *J. Dairy Sci.* 91, 4765–4777. doi:10.3168/jds.2008-1338.

Benchaar, C., Petit, H. V., Berthiaume, R., Ouellet, D. R., Chiquette, J., and Chouinard, P. Y. (2007). Effects of Essential Oils on Digestion, Ruminal Fermentation, Rumen Microbial Populations, Milk Production, and Milk Composition in Dairy Cows Fed Alfalfa Silage or Corn Silage. *J. Dairy Sci.* 90, 886–897. doi:10.3168/jds.S0022-0302(07)71572-2.

Benchaar, C., Petit, H. V., Berthiaume, R., Whyte, T. D., and Chouinard, P. Y. (2006). Effects of Addition of Essential Oils and Monensin Premix on Digestion, Ruminal Fermentation, Milk Production, and Milk Composition in Dairy Cows. *J. Dairy Sci.* 89, 4352–4364. doi:10.3168/jds.S0022-0302(06)72482-1.

Bird, S. H., Hegarty, R. S., and Woodgate, R. (2008). Persistence of defaunation effects on digestion and methane production in ewes. *Aust. J. Exp. Agric.* 48, 152. doi:10.1071/EA07298.

Bryszak, M., Szumacher-Strabel, M., El-Sherbiny, M., Stochmal, A., Oleszek, W., Roj, E., et al. (2019). Effects of berry seed residues on ruminal fermentation, methane concentration, milk production, and fatty acid proportions in the rumen and milk of dairy cows. *J. Dairy Sci.* 102, 1257–1273. doi:10.3168/jds.2018-15322.

Cardozo, P. W., Calsamiglia, S., Ferret, A., and Kamel, C. (2006). Effects of alfalfa extract, anise, capsicum, and a mixture of cinnamaldehyde and eugenol on ruminal fermentation and protein degradation in beef heifers fed a high-concentrate diet1. *J. Anim. Sci.* 84, 2801–2808. doi:10.2527/jas.2005-593.

Carulla, J. E., Kreuzer, M., Machmüller, A., and Hess, H. D. (2005). Supplementation of Acacia mearnsii tannins decreases methanogenesis and urinary nitrogen in forage-fed sheep. *Aust. J. Agric. Res.* 56, 961. doi:10.1071/AR05022.

Chung, Y.-H., He, M. L., McGinn, S. M., McAllister, T. A., and Beauchemin, K. A. (2011). Linseed suppresses enteric methane emissions from cattle fed barley silage, but not from those fed grass hay. *Anim. Feed Sci. Technol.* 166–167, 321–329. doi:10.1016/j.anifeedsci.2011.04.022.

Cieslak, A., Zmora, P., Pers-Kamczyc, E., and Szumacher-Strabel, M. (2012). Effects of tannins source (Vaccinium vitis idaea L.) on rumen microbial fermentation in vivo. *Anim. Feed Sci. Technol.* 176, 102–106. doi:10.1016/j.anifeedsci.2012.07.012.

Faciola, A. P., and Broderick, G. A. (2013). Effects of feeding lauric acid on ruminal protozoa numbers, fermentation, and digestion and on milk production in dairy cows1. *J. Anim. Sci.* 91, 2243–2253. doi:10.2527/jas.2012-5169.

Faciola, A. P., and Broderick, G. A. (2014). Effects of feeding lauric acid or coconut oil on ruminal protozoa numbers, fermentation pattern, digestion, omasal nutrient flow, and milk production in dairy cows. *J. Dairy Sci.* 97, 5088–5100. doi:10.3168/jds.2013-7653.

Faciola, A. P., Broderick, G. A., Hristov, A., and Leão, M. I. (2013). Effects of lauric acid on ruminal protozoal numbers and fermentation pattern and milk production in lactating dairy cows1. *J. Anim. Sci.* 91, 363–373. doi:10.2527/jas.2012-5168.

Fandiño, I., Calsamiglia, S., Ferret, A., and Blanch, M. (2008). Anise and capsicum as alternatives to monensin to modify rumen fermentation in beef heifers fed a high concentrate diet. *Anim. Feed Sci. Technol.* 145, 409–417. doi:10.1016/j.anifeedsci.2007.04.018.

Grainger, C., Williams, R., Clarke, T., Wright, A.-D. G., and Eckard, R. J. (2010). Supplementation with whole cottonseed causes long-term reduction of methane emissions from lactating dairy cows offered a forage and cereal grain diet. *J. Dairy Sci.* 93, 2612–2619. doi:10.3168/jds.2009-2888.

Guyader, J., Eugène, M., Doreau, M., Morgavi, D. P., Gérard, C., and Martin, C. (2017). Tea saponin reduced methanogenesis in vitro but increased methane yield in lactating dairy cows. *J. Dairy Sci.* 100, 1845–1855. doi:10.3168/jds.2016-11644.

H. D. Hess, Beuret, R. A., M. Lötscher, Hindrichsen, I. K., A. Machmüller, Carulla, J. E., et al. (2004). Ruminal fermentation, methanogenesis and nitrogen utilization of sheep receiving tropical grass hay-concentrate diets offered with Sapindus saponaria fruits and Cratylia argentea foliage. *Anim. Sci.* 79, 177–189. doi:10.1017/S1357729800054643.

Hegarty, R. S., Bird, S. H., Vanselow, B. A., and Woodgate, R. (2008). Effects of the absence of protozoa from birth or from weaning on the growth and methane production of lambs. *Br. J. Nutr.* 100, 1220–1227. doi:10.1017/S0007114508981435.

Holtshausen, L., Chaves, A. V., Beauchemin, K. A., McGinn, S. M., McAllister, T. A., Odongo, N. E., et al. (2009). Feeding saponin-containing Yucca schidigera and Quillaja saponaria to decrease enteric methane production in dairy cows. *J. Dairy Sci.* 92, 2809–2821. doi:10.3168/jds.2008-1843.

Hristov, A. N., Grandeen, K. L., Ropp, J. K., and McGuire, M. A. (2004). Effect of Sodium Laurate on Ruminal Fermentation and Utilization of Ruminal Ammonia Nitrogen for Milk Protein Synthesis in Dairy Cows. *J. Dairy Sci.* 87, 1820–1831. doi:10.3168/jds.S0022-0302(04)73339-1.

Hristov, A. N., Lee, C., Cassidy, T., Long, M., Heyler, K., Corl, B., et al. (2011). Effects of lauric and myristic acids on ruminal fermentation, production, and milk fatty acid composition in lactating dairy cows. *J. Dairy Sci.* 94, 382–395. doi:10.3168/jds.2010-3508.

Hristov, A. N., Vander Pol, M., Agle, M., Zaman, S., Schneider, C., Ndegwa, P., et al. (2009). Effect of lauric acid and coconut oil on ruminal fermentation, digestion, ammonia losses from manure, and milk fatty acid composition in lactating cows. *J. Dairy Sci.* 92, 5561–5582. doi:10.3168/jds.2009-2383.

Ivan M, Neill L, Forster R, Alimon R, Rode LM, E. T. (2000). Effects of Isotricha, Dasytricha, Entodinium, and total fauna on ruminal fermentation and duodenal flow in wethers fed different diets. *J Dairy Sci* 83, 776787.

Ivan, M., Petit, H. V., Chiquette, J., and Wright, A.-D. G. (2013). Rumen fermentation and microbial population in lactating dairy cows receiving diets containing oilseeds rich in C-18 fatty acids. *Br. J. Nutr.* 109, 1211–1218. doi:10.1017/S0007114512003030.

Jordan, E., Kenny, D., Hawkins, M., Malone, R., Lovett, D. K., and O’Mara, F. P. (2006a). Effect of refined soy oil or whole soybeans on intake, methane output, and performance of young bulls1. *J. Anim. Sci.* 84, 2418–2425. doi:10.2527/jas.2005-354.

Jordan, E., Lovett, D. K., Monahan, F. J., Callan, J., Flynn, B., and O’Mara, F. P. (2006b). Effect of refined coconut oil or copra meal on methane output and on intake and performance of beef heifers1. *J. Anim. Sci.* 84, 162–170. doi:10.2527/2006.841162x.

Klevenhusen, F., Zeitz, J. O., Duval, S., Kreuzer, M., and Soliva, C. R. (2011). Garlic oil and its principal component diallyl disulfide fail to mitigate methane, but improve digestibility in sheep. *Anim. Feed Sci. Technol.* 166–167, 356–363. doi:10.1016/j.anifeedsci.2011.04.071.

Koenig, K. M., Newbold, C. J., McIntosh, F. M., and Rode, L. M. (2000). Effects of protozoa on bacterial nitrogen recycling in the rumen. *J. Anim. Sci.* 78, 2431. doi:10.2527/2000.7892431x.

Lee, C., Hristov, A. N., Heyler, K. S., Cassidy, T. W., Long, M., Corl, B. A., et al. (2011). Effects of dietary protein concentration and coconut oil supplementation on nitrogen utilization and production in dairy cows. *J. Dairy Sci.* 94, 5544–5557. doi:10.3168/jds.2010-3889.

Lila, Z. A., Mohammed, N., Kanda, S., Kurihara, M., and Itabashi, H. (2005). Sarsaponin Effects on Ruminal Fermentation and Microbes, Methane Production, Digestibility and Blood Metabolites in Steers. *Asian-Australasian J. Anim. Sci.* 18, 1746–1751. doi:10.5713/ajas.2005.1746.

Lovett, D., Lovell, S., Stack, L., Callan, J., Finlay, M., Conolly, J., et al. (2003). Effect of forage/concentrate ratio and dietary coconut oil level on methane output and performance of finishing beef heifers. *Livest. Prod. Sci.* 84, 135–146. doi:10.1016/j.livprodsci.2003.09.010.

Machmüller, A., Ossowski, D. ., and Kreuzer, M. (2000). Comparative evaluation of the effects of coconut oil, oilseeds and crystalline fat on methane release, digestion and energy balance in lambs. *Anim. Feed Sci. Technol.* 85, 41–60. doi:10.1016/S0377-8401(00)00126-7.

Machmüller, A., Soliva, C. R., and Kreuzer, M. (2003a). Effect of coconut oil and defaunation treatment on methanogenesis in sheep. *Reprod. Nutr. Dev.* 43, 41–55. doi:10.1051/rnd:2003005.

Machmüller, A., Soliva, C. R., and Kreuzer, M. (2003b). Methane-suppressing effect of myristic acid in sheep as affected by dietary calcium and forage proportion. *Br. J. Nutr.* 90, 529–540. doi:10.1079/BJN2003932.

Martin, C., Pomiès, D., Ferlay, A., Rochette, Y., Martin, B., Chilliard, Y., et al. (2011). Methane output and rumen microbiota in dairy cows in response to long-term supplementation with linseed or rapeseed of grass silage- or pasture-based diets. *Proc. New Zeal. Soc. Anim. Prod.* 71, 243–247.

Moate, P. J., Williams, S. R. O., Hannah, M. C., Eckard, R. J., Auldist, M. J., Ribaux, B. E., et al. (2013). Effects of feeding algal meal high in docosahexaenoic acid on feed intake, milk production, and methane emissions in dairy cows. *J. Dairy Sci.* 96, 3177–3188. doi:10.3168/jds.2012-6168.

Moate, P. J., Williams, S. R. O., Torok, V. A., Hannah, M. C., Ribaux, B. E., Tavendale, M. H., et al. (2014). Grape marc reduces methane emissions when fed to dairy cows. *J. Dairy Sci.* 97, 5073–5087. doi:10.3168/jds.2013-7588.

Mohammed, N., Ajisaka, N., Lila, Z. A., Hara, K., Mikuni, K., Hara, K., et al. (2004). Effect of Japanese horseradish oil on methane production and ruminal fermentation in vitro and in steers1. *J. Anim. Sci.* 82, 1839–1846. doi:10.2527/2004.8261839x.

Morgavi, D. P., Jouany, J.-P., and Martin, C. (2008). Changes in methane emission and rumen fermentation parameters induced by refaunation in sheep. *Aust. J. Exp. Agric.* 48, 69. doi:10.1071/EA07236.

Morgavi, D. P., Martin, C., Jouany, J.-P., and Ranilla, M. J. (2012). Rumen protozoa and methanogenesis: not a simple cause–effect relationship. *Br. J. Nutr.* 107, 388–397. doi:10.1017/S0007114511002935.

Newbold, C. ., McIntosh, F. ., Williams, P., Losa, R., and Wallace, R. . (2004). Effects of a specific blend of essential oil compounds on rumen fermentation. *Anim. Feed Sci. Technol.* 114, 105–112. doi:10.1016/j.anifeedsci.2003.12.006.

Nguyen, S. H., and Hegarty, R. S. (2017). Effects of defaunation and dietary coconut oil distillate on fermentation, digesta kinetics and methane production of Brahman heifers. *J. Anim. Physiol. Anim. Nutr. (Berl).* 101, 984–993. doi:10.1111/jpn.12534.

OZUTSUMI, Y., TAJIMA, K., TAKENAKA, A., and ITABASHI, H. (2005). The Effect of Protozoa on the Composition of Rumen Bacteria in Cattle Using 16S rRNA Gene Clone Libraries. *Biosci. Biotechnol. Biochem.* 69, 499–506. doi:10.1271/bbb.69.499.

Panyakaew, P., Boon, N., Goel, G., Yuangklang, C., Schonewille, J. T., Hendriks, W. H., et al. (2013). Effect of supplementing coconut or krabok oil, rich in medium-chain fatty acids on ruminal fermentation, protozoa and archaeal population of bulls. *animal* 7, 1950–1958. doi:10.1017/S1751731113001766.

Pen, B., Takaura, K., Yamaguchi, S., Asa, R., and Takahashi, J. (2007). Effects of Yucca schidigera and Quillaja saponaria with or without β 1–4 galacto-oligosaccharides on ruminal fermentation, methane production and nitrogen utilization in sheep. *Anim. Feed Sci. Technol.* 138, 75–88. doi:10.1016/j.anifeedsci.2006.11.018.

Pilajun, R., and Wanapat, M. (2011). Effect of coconut oil and mangosteen peel supplementation on ruminal fermentation, microbial population, and microbial protein synthesis in swamp buffaloes. *Livest. Sci.* 141, 148–154. doi:10.1016/j.livsci.2011.05.013.

Reveneau, C., Karnati, S. K. R., Oelker, E. R., and Firkins, J. L. (2012). Interaction of unsaturated fat or coconut oil with monensin in lactating dairy cows fed 12 times daily. I. Protozoal abundance, nutrient digestibility, and microbial protein flow to the omasum. *J. Dairy Sci.* 95, 2046–2060. doi:10.3168/jds.2011-4887.

Rira, M., Morgavi, D. P., Archimède, H., Marie-Magdeleine, C., Popova, M., Bousseboua, H., et al. (2015). Potential of tannin-rich plants for modulating ruminal microbes and ruminal fermentation in sheep1. *J. Anim. Sci.* 93, 334–347. doi:10.2527/jas.2014-7961.

Sahoo, A., Kamra, D. N., and Pathak, N. N. (2005). Pre- and Postweaning Attributes in Faunated and Ciliate-Free Calves Fed Calf Starter With or Without Fish Meal. *J. Dairy Sci.* 88, 2027–2036. doi:10.3168/jds.S0022-0302(05)72879-4.

Santra, A., and Karim, S. A. (2000). Growth performance of faunated and defaunated Malpura weaner lambs. *Anim. Feed Sci. Technol.* 86, 251–260. doi:10.1016/S0377-8401(00)00161-9.

Schönhusen, U., Zitnan, R., Kuhla, S., Jentsch, W., Derno, M., and Voigt, J. (2003). Effects of protozoa on Methane production in rumen and hindgut of calves around time of weaning. *Arch. Anim. Nutr.* 57, 279–295. doi:10.1080/00039420310001594423.

Soltan, M. A. E.-K., Shewita, R. S., and Al-Sultan, S. I. (2009). Influence of Essential Oils Supplementation on Digestion, Rumen Fermentation, Rumen Microbial Populations and Productive Performance of Dairy Cows. *Asian J. Anim. Sci.* 3, 1–12. doi:10.3923/ajas.2009.1.12.

Staerfl, S. M., Zeitz, J. O., Kreuzer, M., and Soliva, C. R. (2012). Methane conversion rate of bulls fattened on grass or maize silage as compared with the IPCC default values, and the long-term methane mitigation efficiency of adding acacia tannin, garlic, maca and lupine. *Agric. Ecosyst. Environ.* 148, 111–120. doi:10.1016/j.agee.2011.11.003.

Tekippe, J. A., Hristov, A. N., Heyler, K. S., Cassidy, T. W., Zheljazkov, V. D., Ferreira, J. F. S., et al. (2011). Rumen fermentation and production effects of Origanum vulgare L. leaves in lactating dairy cows. *J. Dairy Sci.* 94, 5065–5079. doi:10.3168/jds.2010-4095.

Yáñez-Ruiz, D. R., Williams, S., and J. Newbold, C. (2007). The effect of absence of protozoa on rumen biohydrogenation and the fatty acid composition of lamb muscle. *Br. J. Nutr.* 97, 938–948. doi:10.1017/S0007114507675187.

Yang, W. Z., Ametaj, B. N., Benchaar, C., and Beauchemin, K. A. (2010a). Dose response to cinnamaldehyde supplementation in growing beef heifers: Ruminal and intestinal digestion1. *J. Anim. Sci.* 88, 680–688. doi:10.2527/jas.2008-1652.

Yang, W. Z., Benchaar, C., Ametaj, B. N., and Beauchemin, K. A. (2010b). Dose response to eugenol supplementation in growing beef cattle: Ruminal fermentation and intestinal digestion. *Anim. Feed Sci. Technol.* 158, 57–64. doi:10.1016/j.anifeedsci.2010.03.019.

Yang, W. Z., Benchaar, C., Ametaj, B. N., Chaves, A. V., He, M. L., and McAllister, T. A. (2007). Effects of Garlic and Juniper Berry Essential Oils on Ruminal Fermentation and on the Site and Extent of Digestion in Lactating Cows. *J. Dairy Sci.* 90, 5671–5681. doi:10.3168/jds.2007-0369.

Zhou, Y. Y., Mao, H. L., Jiang, F., Wang, J. K., Liu, J. X., and McSweeney, C. S. (2011). Inhibition of rumen methanogenesis by tea saponins with reference to fermentation pattern and microbial communities in Hu sheep. *Anim. Feed Sci. Technol.* 166–167, 93–100. doi:10.1016/j.anifeedsci.2011.04.007.

Supplementary Table 2 Statistical description of the diet and animal characteristics in the data set

| Item | Sheep | | | Dairy | | | Beef | | |
| --- | --- | --- | --- | --- | --- | --- | --- | --- | --- |
|  | N | Mean | Std Dev | N | Mean | Std Dev | N | Mean | Std Dev |
| *Diet composition, %* |  |  |  |  |  |  |  |  |  |
| DM | 32 | 67.1 | 26.0 | 45 | 54.5 | 8.15 | 37 | 85.6 | 8.49 |
| OM | 65 | 91.9 | 2.96 | 63 | 91.7 | 4.50 | 27 | 92.3 | 1.53 |
| NDF | 74 | 40.4 | 11.8 | 81 | 32.9 | 4.84 | 44 | 29.1 | 12.6 |
| CP | 70 | 16.7 | 3.87 | 81 | 16.5 | 1.79 | 44 | 16.8 | 4.85 |
| *Ruminal microorganisms, log cells/ mL* |  |  |  |  |  |  |  |  |  |
| Protozoa | 67 | 4.85 | 1.85 | 91 | 5.81 | 0.68 | 47 | 6.44 | 1.57 |
| Bacteria | 27 | 9.70 | 1.07 | 17 | 9.79 | 0.70 | 10 | 9.53 | 0.98 |
| *Methane production, g/kg DMI* | 33 | 28.2 | 10.1 | 36 | 19.9 | 7.20 | 24 | 25.6 | 7.27 |
| *Ruminal fermentation* |  |  |  |  |  |  |  |  |  |
| pH | 65 | 6.50 | 0.35 | 87 | 6.28 | 0.31 | 42 | 6.27 | 0.32 |
| Total VFA concentration, *mM* | 80 | 97.4 | 25.4 | 97 | 108 | 20.9 | 46 | 99.9 | 33.0 |
| Molar proportion, % |  |  |  |  |  |  |  |  |  |
| Acetate | 80 | 68.0 | 4.23 | 97 | 62.3 | 4.45 | 44 | 62.4 | 7.32 |
| Propionate | 80 | 19.8 | 3.62 | 97 | 21.8 | 3.22 | 44 | 22.2 | 5.42 |
| Butyrate | 80 | 9.35 | 2.88 | 97 | 11.7 | 2.07 | 44 | 11.6 | 2.59 |
| NH_3_N, *mM* | 61 | 10.1 | 6.23 | 81 | 7.27 | 3.57 | 42 | 7.88 | 4.84 |
| *Total tract digestibility, %* |  |  |  |  |  |  |  |  |  |
| DM | 19 | 65.8 | 7.20 | 60 | 64.3 | 4.17 | 18 | 60.8 | 9.06 |
| OM | 61 | 67.4 | 6.62 | 56 | 66.4 | 3.97 | 19 | 69.8 | 7.25 |
| CP | 33 | 70.0 | 13.3 | 47 | 63.4 | 4.89 | 18 | 66.0 | 4.12 |
| NDF | 49 | 54.0 | 8.70 | 55 | 45.9 | 9.42 | 27 | 54.5 | 7.67 |
| *Animal performance* |  |  |  |  |  |  |  |  |  |
| Milk yield, kg/d | - | - | - | 78 | 29.7 | 6.08 | - | - | - |
| Milk composition, % |  |  |  |  |  |  |  |  |  |

Supplementary Table 2. Continued.

| Item | Sheep | | | Dairy | | | Beef | | |
| --- | --- | --- | --- | --- | --- | --- | --- | --- | --- |
|  | N | Mean | Std Dev | N | Mean | Std Dev | N | Mean | Std Dev |
| Protein | - | - | - | 72 | 3.31 | 0.41 | - | - | - |
| Fat | - | - | - | 72 | 3.53 | 0.55 | - | - | - |
| Lactose | - | - | - | 70 | 4.70 | 0.17 | - | - | - |
| DMI, kg/d | 46 | 0.93 | 0.26 | 83 | 20.3 | 3.85 | 46 | 7.06 | 2.17 |
| BWG, kg | 7 | 3.34 | 5.48 | 30 | 3.83 | 8.01 | 25 | 2.40 | 4.44 |

Supplementary Table 3 Summary of the meta-analysis.

| Items^1^ | Treatment | | | Factor^2^ | | |
| --- | --- | --- | --- | --- | --- | --- |
|  | Complete | Phytochemicals | Lipids | Trt | RP | Trt x RP |
| *Ruminal protozoa, Log10 cells/mL* | - | NS | ↓ | - | - | - |
| *Ruminal Bacteria, Log10 cells/ mL* | ↑ | NS | NS |  | ▲ |  |
| *Methane production, g/kg DMI* | ↓ | ↓ | ↓ |  |  | ▲ |
| *Ruminal fermentation* |  |  |  |  |  |  |
| pH | NS | NS | NS |  | ▲ |  |
| Total VFA concentration, *mM* | ↓ | NS | ↓ |  | △ |  |
| Molar proportion, % |  |  |  |  |  |  |
| Acetate | ↑ | ↓ | ↓ |  |  | ▲ |
| Propionate | ↑ | ↑ | ↑ |  |  | ▲ |
| Butyrate | ↓ | ↑ | ↓ | ▲ | △ |  |
| NH_3_-N, *mM* | ↓ | ↓ | ↓ | ▲ | ▲ |  |
| *Total tract digestibility, %* |  |  |  |  |  |  |
| DM | NS | NS | ↓ | NS | NS | NS |
| OM | ↓ | ↓ | ↓ | ▲ | △ |  |
| CP | NS | NS | NS | NS | NS | NS |
| NDF | ↓ | ↓ | ↓ |  |  | ▲ |
| *Animal performance* |  |  |  |  |  |  |
| Milk yield, kg/d | − | NS | ↓ |  | ▲ |  |
| Milk composition, % |  |  |  |  |  |  |
| Protein | − | ↓ | ↓ | ▲ |  |  |
| Fat | − | NS | ↓ |  | ▲ |  |
| Lactose | − | NS | ↓ | NS | NS | NS |
| DMI, kg/d |  |  |  |  |  |  |
| Sheep | NS | ↑ | NS |  |  | ▲ |
| Dairy | − | ↓ | ↓ | ▲ | ▲ |  |
| Beef | NS | NS | NS | NS | NS | NS |
| BWG, kg | NS | NS | NS | NS | NS | NS |

^1^VFA = voile fatty acids; DM = dry matter; OM = Organic matter; CP = crude protein; NDF = neutral detergent fiber; BWG = body weight gain.

^2^ Data only considering the partial RP elimination (phytochemicals and lipids); trt = treatment; RP= ruminal protozoa.

↑ = increased, ↓ =decreased; NS= No significant; − =No present; ▲= significant effect; △ = significant effects and RP affected the effects of treatment on the response variable of interest.
